# Synthesis, FT-IR, structural, thermochemical, electronic absorption spectral, and NLO analysis of the novel 10-methoxy-10*H*-furo[3,2-*g*]chromeno[2,3-*b*][1,3]thiazolo[5,4-*e*]pyridine-2,10(3*H*)-dione (MFCTP): a DFT/TD-DFT study

**DOI:** 10.1039/d1ra06134h

**Published:** 2021-09-29

**Authors:** Shimaa Abdel Halim, Magdy A. Ibrahim

**Affiliations:** Department of Chemistry, Faculty of Education, Ain Shams University Roxy 11711 Cairo Egypt shimaaquantum@ymail.com +20 01090306455

## Abstract

Chemical transformation of 4-methoxy-5-oxo-5*H*-furo[3,2-*g*]chromene-6-carbonitrile (1) with 1,3-thiazolidine-2,4-dione (2) in boiling ethanol containing piperidine afforded the novel 10-methoxy-10*H*-furo[3,2-*g*]chromeno[2,3-*b*][1,3]thiazole[5,4-*e*]pyridine-2,10(3*H*)-dione (3, MFCTP). The chemical structure of the synthesized compound was established *via* elemental analysis and spectral data. FT-IR spectroscopy was performed in the range of 400–4000 cm^−1^ for the vibrational spectral analysis of MFCTP. The GIAO method was employed to calculate the values of ^1^H and ^13^C NMR chemical shifts theoretically, which were consistent with the experimental chemical shifts. The molecule (3, MFCTP) has two stable structures, as determined from the potential energy curve. The S1 structure is the most stable conformer of (3, MFCTP) according to the computational results. The density functional theory (DFT) and *ab initio* HF calculations and different basis set combinations based on the structure optimizations and normal coordinate force field were interpreted with the aid of the molecular structure, fundamental vibrational frequencies, and intensities of the vibrational bands. The potential energy distribution (PED) was determined based on the complete vibrational wavenumber assignments. The calculated spectra of the title compound were in agreement with the observed spectra. The scaled B3LYP/6-311++G(d,p) results exhibited better agreement with the experimental values compared to the other method used. The time-dependent density functional theory (TD-DFT) was employed to calculate the energy and oscillator strength and supplement the experimental findings. Also, it was performed and the results interpreted the molecular electrostatic potential, nonlinear optical and thermodynamic properties, and Mulliken and natural charges of the title compound. DFT calculations were performed to study the structure–activity relationship (SAR) and compared with the experimental antimicrobial results for compound (3, MFCTP).

## Abstract 2

A novel MFCTP was synthesized and characterized.

## Graphical abstract

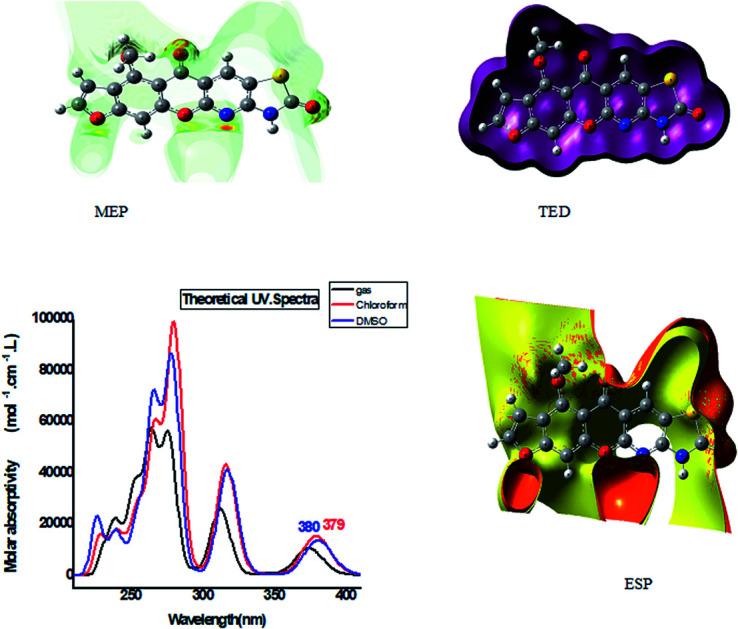

## Introduction

Khellin and visnagin are naturally occurring furochromone derivatives extracted from the seeds of *Ammi visnaga* L.^1^ They are broadly used as anti-inflammatory, analgesic,^2,3^ anticancer,^4,5^ anticonvulsant,^6^ antitubercular^7^ and antimicrobial agents.^8^ The optimized geometries of some furo[3,2-*g*]chromenes have been examined by DFT-theoretical calculations.^9,10^ Photoelectrical, photosensitivity, photovoltaic, photodiode, electronic spectra, solvatochromic computational and molecular docking studies have been performed on a range of furo[3,2-*g*]chromene derivatives.^11–16^ The experimental values of the molecular geometry, vibrational frequencies, atomic charges, dipole moment, thermodynamical properties, *etc.* can be reproduced *via* DFT and computational methods, which are employed to calculate the electronic structure of molecular systems because of their high accuracy.^17–22^ However, in the literature, there is no published work regarding experimental and computational vibrational spectroscopic data on compound (3, MFCTP), therefore; this inspired us to investigate its FT-IR and ^1^H, ^13^C NMR spectra theoretically and experimentally. The current work presents the spectroscopic, thermodynamic properties and electronic structure of compound (3, MFCTP). In continuation of our previous work on heterocyclic compounds,^23–25^ the current study aimed to examine the properties of compound (3, MFCTP) and predict its applications. We provide a broad description of the chemical reactivity of compound (3, MFCTP) based on the analysis of its bond natural orbital (NBO) charge delocalization and chemical shift (NMR), FT-IR, and vibrational data. Its thermodynamic properties were also calculated at the B3LYP/6-311G (d,p) theoretical level. The molecular modeling study with hybrid quantum mechanics on the title compound is not available. The structure and binding properties are reported for the first time in this study. The electronic properties of compound (3, MFCTP) such as its HOMO–LUMO energy gap, chemical hardness, and chemical potential were calculated. In addition, the molecular electrostatic potential (MEP) and UV-visible spectra of compound (3, MFCTP) were studied using theoretical calculations and experiments to provide its spectra and electronic structure using CAM-B3LYP/6-311++G(d,p). The non-linear optical (NLO) parameters were calculated at the same level of theory, including the electronic dipole moment (*μ*), first-order hyperpolarizability (*β*), hyper-Rayleigh scattering (*β*_HRS_), and depolarization ratio (DR). All calculations in this research were performed using the DFT method at the B3LYP/6-311++G(d,p) level of theory. Also, the structure–activity relationship (SAR) was studied for the antimicrobial application of the current compound (3, MFCTP).

## Experimental

### 10-Methoxy-10*H*-furo[3,2-*g*]chromeno[2,3-*b*][1,3]thiazolo[5,4-*e*]pyridine-2,10(3*H*)-dione (3, MFCTP)

A mixture of 4-methoxy-5-oxo-5*H*-furo[3,2-*g*] chromene-6-carbonitrile (1) (0.48 g, 2 mmol) and 1,3-thiazolidine-2,4-dione (2) (0.23 g, 2 mmol) in absolute ethanol (20 mL) containing piperidine (0.1 mL) was heated under reflux for 2 h. The yellow crystals formed during heating were filtered and recrystallized from *n*-butanol to give compound (3, MFCTP), yield (0.57 g, 81%), mp 296–297 °C. IR (KBr, cm^−1^): 3225 (NH), 3122 (CH_furan_), 3055 (CH_arom_), 2948, 2841 (CH_aliph_), 1679 (C=O_cyclic amide_), 1638 (C=O_γ-pyrone_), 1568 (C=C). ^1^H-NMR (300 MHz, DMSO-*d*_6_, *δ*): 3.96 (s, 3H, OCH_3_), 7.09 (s, 1H, H-6), 7.16 (d, 1H, *J* = 2.4 Hz, H-3_furan_), 7.92 (d, 1H, *J* = 2.4 Hz, H-2_furan_), 8.87 (s, 1H, H-12), 11.40 (bs, 1H, NH exchangeable with D_2_O). ^13^C-NMR (100 MHz, DMSO-*d*_6_, *δ*): 61.3 (OMe), 93.0 (C-6), 105.9 (C-3_furan_), 107.3 (C-10a), 110.4 (C-9a), 114.3 (C-11a), 117.9 (C-12a), 131.9 (C-12), 146.5 (C-2_furan_), 152.6 (C-5a), 153.9 (C-4), 154.8 (C-4a), 158.4 (C-3a), 162.6 (C-6a), 171.8 (C-2 as C=O), 176.8 (C-5 as C=O). Mass spectrum, *m*/*z* (*I*_r_%): 340 (M^+^, 100), 312 (62), 296 (43), 269 (58), 191 (41), 175 (28), 169 (12), 147 (34), 123 (9), 118 (70), 107 (40), 91 (23), 77 (27), 64 (13). Anal. calcd for C_16_H_8_N_2_O_5_S (340.31): C, 56.47%; H, 2.37%; N, 8.23%; S, 9.42%. Found: C, 56.25%; H, 2.20%; N, 8.15%; S, 9.30%.

### Instruments

Melting points were measured using a digital Stuart SMP3 apparatus. Infrared spectra measured using a Nicolet IS10 FTIR spectrophotometer (cm^−1^) and KBr disks. ^1^H NMR (300 MHz) and ^13^C NMR (100 MHz) spectra were measured using a Mercury-300BB apparatus, with TMS (*δ*) as the internal standard and DMSO-*d*_6_ as the solvent. Mass spectra were measured using a GC-2010 Shimadzu gas chromatography instrument mass spectrometer (70 eV). Elemental microanalyses were performed at the Chemical War Department, Ministry of Defense, Egypt using a PerkinElmer 2400II. Thin-layer chromatography and elemental microanalysis were used to check the purity of the synthesized compounds. Also, a PerkinElmer Lambda 4B spectrophotometer was used to measure the absorption electronic spectra in the wavelength range of 200–900 nm using 1.0 cm fused quartz cells.

### Solvents

Merck, AR-grade DMSO as the polar solvent and chloroform as the non-polar solvent were used without purification.

### Antimicrobial study

The biological activity of the synthesized compound (3, MFCTP) was examined for its antibacterial and antifungal properties against different types of bacteria, including the Gram-positive *S. aureus* and *B. subtilis* and Gram-negative *S. typhimurium* and *E. coli*, and also, yeast *C. albicans* and fungus *A. fumigatus*.

### Computational details

The Gaussian 09 program^26^ was used for the quantum chemical calculations using the *ab initio* HF and DFT (B3LYP) methods with the 6-311++G(d,p) basis set. The vibrational frequencies were calculated from the optimized structural parameters at different level of theories and assuming *C*_1_ point group symmetry. The frequency modes of the title molecule (MFCTP) not acquired at the optimized geometry were determined from a true minimum on the potential energy surface. Based on this, the unscaled calculated frequencies, reduced masses, force constants, infrared intensities, Raman intensities, and depolarization ratios were obtained. To improve the calculated values a spectral uniform scaling factor was employed, which decreased the systematic errors caused by basis set incompleteness, neglecting electron correlation and vibrational anharmonicity, in agreement with the experimental values. Consequently, the calculated frequency vibration at the HF level was scaled by 0.905 (ref. 27) and wavenumbers above and below 1700 cm^−1^ were scaled by 0.958 and 0.983, respectively, for B3LYP/6-311++G(d,p).^28,29^ The deviation from the experiments was less than 10 cm^−1^ after scaling with the scaling factor, with a few exceptions. PEDs were based on the assignments of the calculated normal modes. The PEDs were computed from quantum chemically calculated vibrational frequencies using the Hyper-Chem Release 8.0 program. Gauss View 5.0 (ref. 30) or Chem Craft 1.6 (ref. 31) was employed for visual animation and verification of the normal mode assignments.

The optimized electronic absorption spectra of (3, MFCTP) are discussed and its reactive sites and molecular electrostatic potential estimated, at the TD-DFT/B3LYP/6-311++G(d,p) level. Also, some theoretical calculations were obtained from molecular polarizabilities such as the dipole moment, linear polarizability and first hyperpolarizability for nonlinear optics (NLO). In addition, the vibrational frequency calculations were performed at different temperatures to show the changes in the thermodynamic functions (heat capacity, entropy, and enthalpy) of (3, MFCTP).

## Results and discussion

### Chemistry

**Scheme 1:**
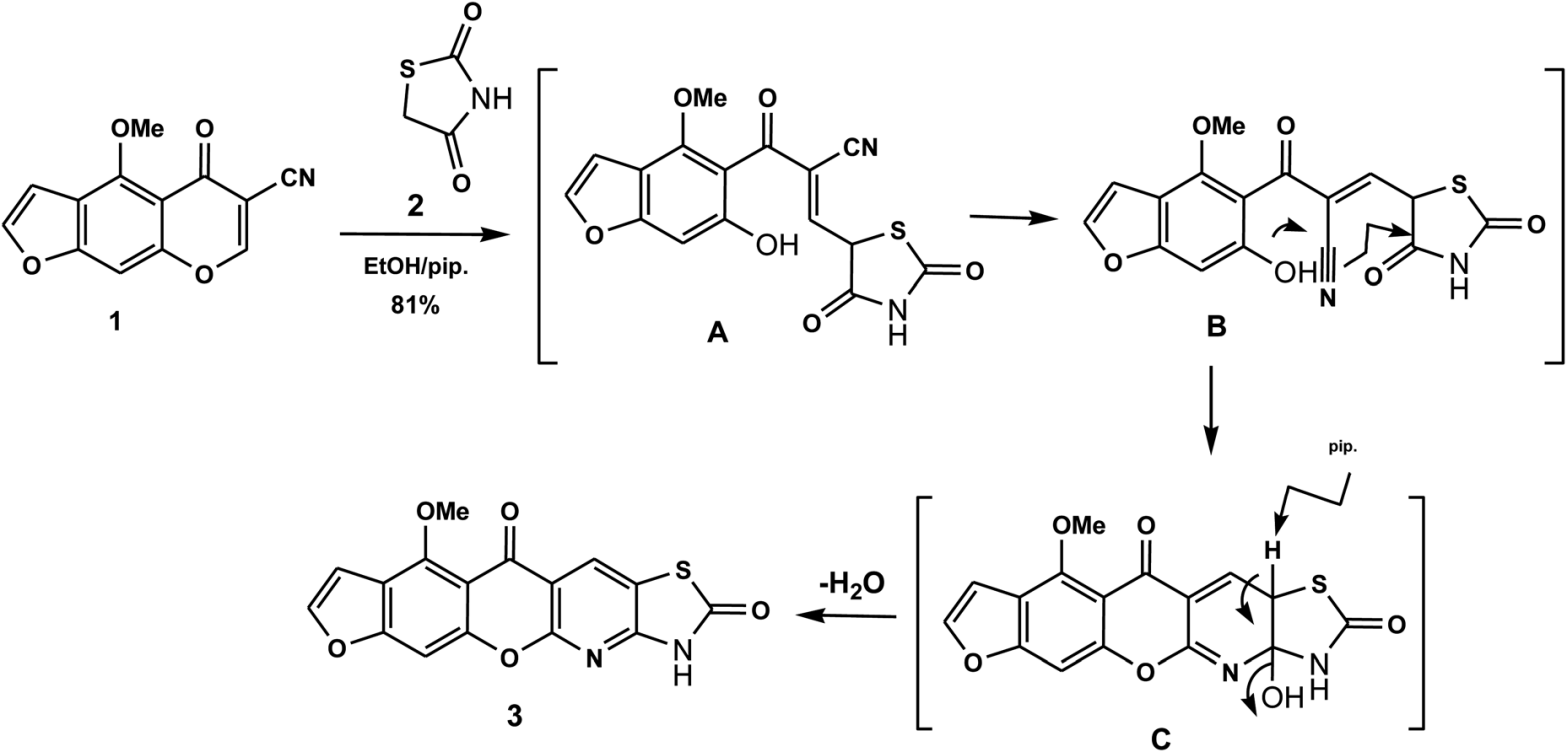
Suggested mechanism for the formation of compound (3, MFCTP).

**Fig. 1:**
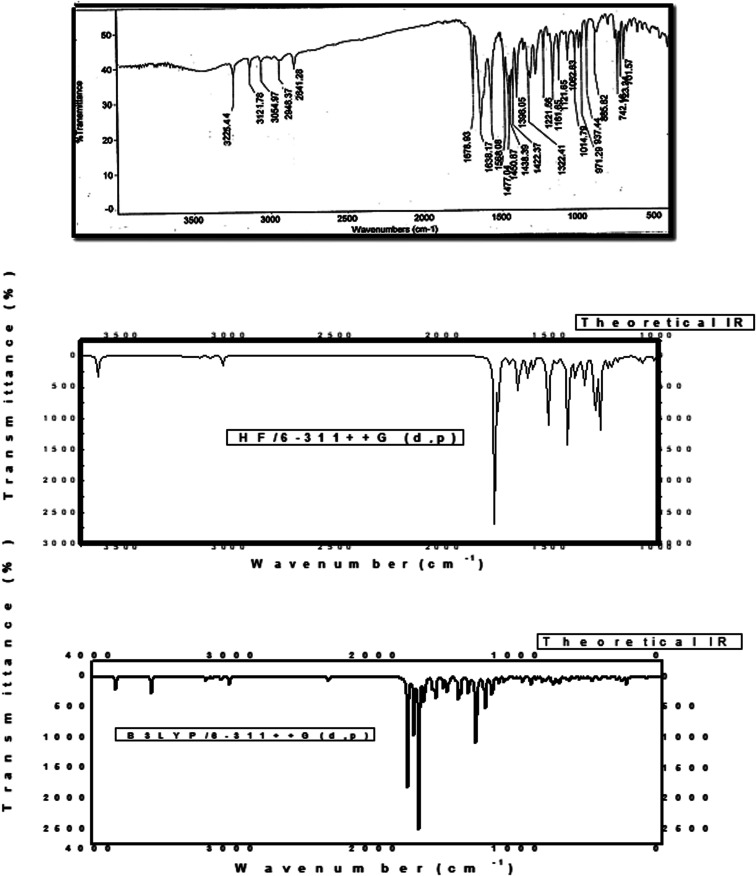
Experimental and calculated IR spectra of compound (3, MFCTP) at the B3LYP/6-311++G(d,p) level.

**Fig. 2:**
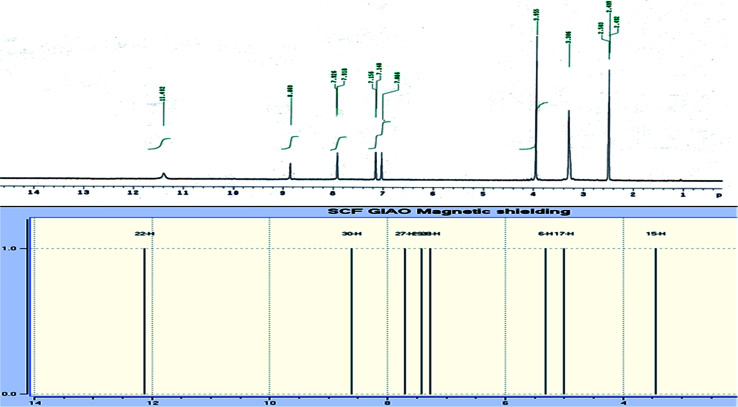
Experimental and calculated ^1^H NMR spectrum of compound (3, MFCTP) at the B3LYP/6-311++G(d,p) level.

**Fig. 3:**
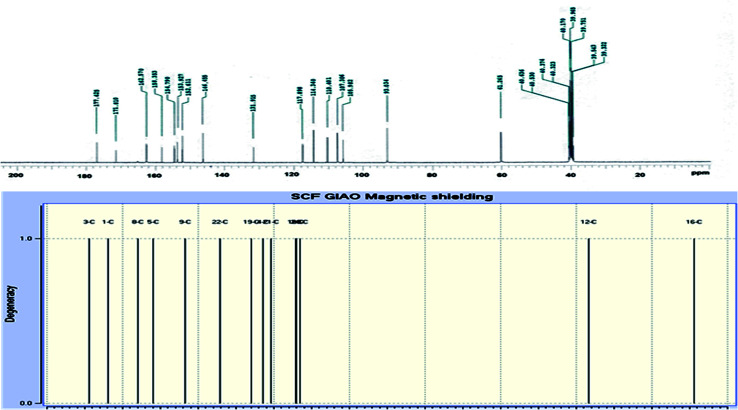
Experimental and calculated ^13^C NMR spectrum of compound (3, MFCTP) at the B3LYP/6-311++G(d,p) level.

**Fig. 4:**
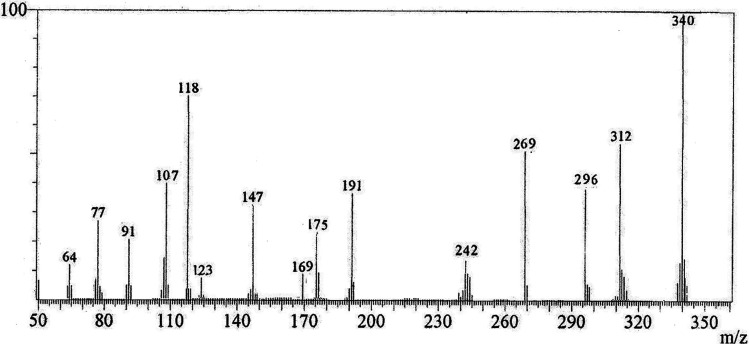
Mass spectrum of compound (3, MFCTP).

**Scheme 2:**
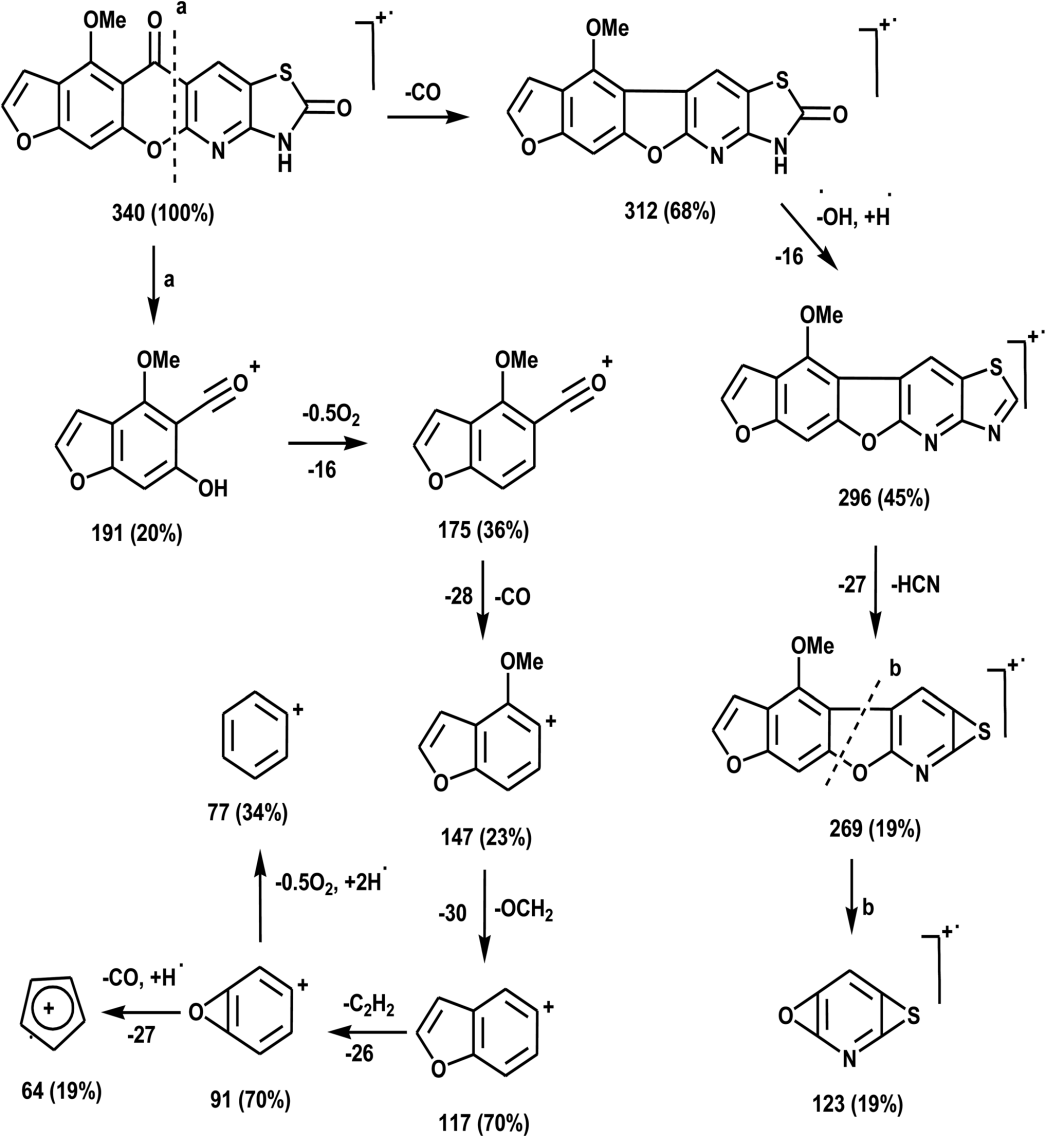
Mass fragmentation patterns of compound (3, MFCTP).

The novel 10-methoxy-10*H*-furo[3,2-*g*]chromeno[2,3-*b*][1,3]thiazolo[5,4-*e*]pyridine-2,10(3*H*)-dione (3, MFCTP) was prepared from the reaction of 4-methoxy-5-oxo-5*H*-furo[3,2-*g*]chromene-6-carbonitrile (1)^32^ with 1,3-thiazolidine-2,4-dione (2)^33^ in boiling ethanol containing a few drops of piperidine (Scheme 1).^34^ The formation of the heteroannulated furochromenothiazolopyridine derivative 3 occurs through cascade reactions involving deprotonation of the active methylene group in 1,3-thiazolidinedione (2) followed by nucleophilic attack at C-7 with ring opening to produce intermediate A, which is converted to conformer B. Two consecutive cycloaddition reactions afford intermediate C, which is dehydrated under the reaction conditions, giving the final product 3, as depicted in Scheme 1. The IR spectrum of compound 3 (Fig. 1) shows the typical absorption bands at 3225 cm^−1^ (NH), 3122 cm^−1^ (CH_furan_), 3055 cm^−1^ (CH_arom_), 2948 and 2841 cm^−1^ (CH_aliph_), 1679 cm^−1^ (C=O_cyclic amide_), 1638 cm^−1^ (C=O_γ-pyrone_) and 1568 cm^−1^ (C=C). The ^1^H-NMR spectrum of compound 3 (Fig. 2) consists of three singlet signals attributed to OCH_3_ (*δ* 3.96), H-6 (*δ* 7.09), H-12 (*δ* 8.87), and two doublets (*J* = 2.4 Hz) attributed to H-3_furan_ (*δ* 7.16) and H-2_furan_ (*δ* 7.92), in addition to a D_2_O exchangeable signal assignable to NH at *δ* 11.40 ppm. The ^13^C NMR spectrum of compound 3 (Fig. 3) presents good evidence for the suggested formula and documented sixteen signals, which equal the sum of carbon atoms existing in the produced molecule. These signals were characterized as follows (ppm): 61.3 (OMe), 93.0 (C-6), 105.9 (C-3_furan_), 107.3 (C-10a), 110.4 (C-9a), 114.3 (C-11a), 117.9 (C-12a), 131.9 (C-12), 146.5 (C-2_furan_), 152.6 (C-5a), 153.9 (C-4), 154.8 (C-4a), 158.4 (C-3a), 162.6 (C-6a), 171.8 (C-2 as C=O), and 176.8 (C-5 as C=O). The mass spectrum of compound 3 (Fig. 4) recorded its molecular ion peak, as the base peak, at *m*/*z* 340 and supports the identity of the structure. The mass fragmentation patterns of compound 3 are depicted in Scheme 2.

### NMR analysis

DFT theory in the gas phase and DMSO solvent was employed to calculate the ^1^H and ^13^C NMR chemical shifts of the title molecule theoretically, which were compared with the experimental NMR in DMSO solvent, as shown in Fig. 2 and 3, respectively. Primarily, the full geometry optimization of the molecule (MFCTP) was performed at the gradient-corrected DFT using the hybrid B3LYP method and GIAO.^35–37^ The ^1^H and ^13^C chemical shift calculations of the compound were performed with the same method using the 6-311++G(d,p) basis set in the gas phase and DMSO solvent. According to the computed and experimental chemical shift values, H22, H23, H24 and H25 have smaller values than the proton signals of H31 and H21, which may be attributed to the electronic charge density around the ring. In the experimental ^13^C NMR spectrum (DMSO), the *δ* values (chemical shift) of the carbon atoms are between 61 and 177 ppm. The molecule has fifteen carbons; however, these carbons were differentiated into three groups (attached with fur chromone, pyridine, and thiazole), which are consistent with the structure and molecular symmetry.

### Electronic structures

#### Conformational isomers, potential energy surface (PES) scan, and molecular geometry

**Fig. 5:**
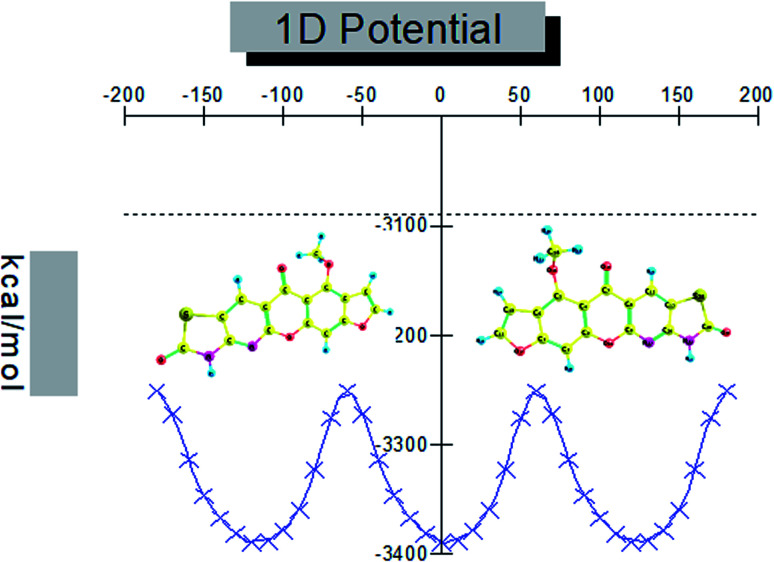
PES scan for the selected degree, (3, MFCTP) torsional freedom.

There is one substituent methoxy group in the studied compound MFCTP joined to five different planar rings. These groups in the compound 3 (MFCTP) molecule were chosen to investigate the structures of its possible conformers. Fig. 5 describes the conformational flexibility of the O–C–N–C torsion angle energy with the AM1 method for the title molecule. The O–C–N–C torsional angle of all the geometrical parameters was changed during the calculations in steps of 10° (*cf.*Fig. 5). For the S1 and S2 conformers, *T*(O–C–N–C) has local minima at 0° (or 360°) and 180° respectively. These results clearly indicate that there are two possible structures for compound 3 (MFCTP), namely, the site of (–CH_3_) fused to (O–CH_3_) is fixed nearer to or further from the ring. The potential energy curve presented in Fig. 5 shows that the S1 formula has the smallest energies. The calculations in this study were performed for two conformers (S1 and S2) of the title molecule, but the tables and figures present further data for only the most stable conformer (S1 form).

**Fig. 6:**
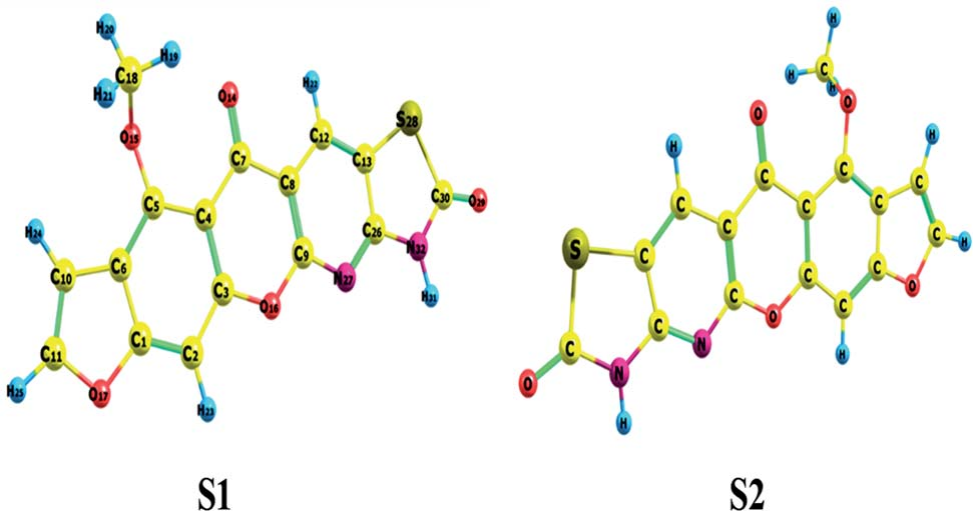
Theoretical possible optimized geometric structures with atom numbering of compound (3, MFCTP).

The hypothetical possible optimized symmetrical configurations including the atom numbering of molecule 3 (MFCTP) are presented in Fig. 6. Table 1 shows the distances, angles, and dihedral angles for the standard internal coordinates, revealing the position of the atoms with respect to a basis atom for 3, MFCTP. The calculations related to the optimized geometries were performed by recreation of all the parameters, which match the real energy minima appearing in the imaginary frequencies of the vibrational mode calculation.

**Table 1:** Definition of the internal symmetry coordinates of compound 3 (MFCTP)

No.	Mode	Vibration	Definition
1	C–H	Stretching	C12H22, C2H23, C10H24, C11H25, C18H19, H20, H21
2	N–H	Stretching	N32H31
3	C–C	Stretching	C13C26, C1C6, C8C9, C10C11,
4	C–N	Stretching	C9N27, C26N27, C30N32, C26N32
5	C–O	Stretching	C7O14, C5O15, C3O16, C1O17
6	C–S	Bending	C13S28, C30S28
7	H–C=N	Bending	C26N27H31,
8	C–C=O	Bending	C3C9O16
9	C–S–C	Bending	C13S28C30
10	C–N–C	Bending	C9N27C26, C26N32C30
11	H–N–C	Bending	N32H31C30
12	C–O–C	Bending	C1O17C11
13	O–C–N	Bending	O16C9N27
14	H–N–C–C	Torsion	N32H31C30C26
15	H–C–C–S	Torsion	H22C12C13S28
16	O–C–N–C	Torsion	O29C30N32C26
17	C–C–S–C	Torsion	C26C13S28C30
18	C–C–O–C	Torsion	C2C3O16C9
19	C–N–C–N	Torsion	C9N27C26N32
20	O–C–S–C	Torsion	O29C30S28C13

The HF and DFT methods with 6-311++G(d,p) were used to calculate the bond lengths and bond angles of the optimized S1 form of compound 3 (MFCTP) (*cf.*Table 2). MFCTP is a planar structure according to the ground state bond angle and dihedral angle calculations and the OCH_3_ group is on the same plane of the ring. B3LYP gave larger bond distances than that from the HF method. There is one N–H, two C–S, four C–N, and eight C–O bonds for MFCTP. The B3LYP/6-311++G(d,p) method overestimated the bond lengths, particularly the C–H bond lengths. The bond length or bond angle of the C–H bond upon substitution resulted in a change in frequency because of the modification on the carbon atom charge division of the ring.^38,39^ The C–H bond length of 1.083 Å was studied in the substituted ring using the B3LYP/6-311++G(d,p) method. In the present work, the ring carbon atoms attract a significant part of the valence electron cloud of the H atom, resulting in an increase in the C–H force constant and a decrease in the corresponding bond length. The shortest C–H bond length was 1.078 Å in the HF/6-311++G(d,p) method. Longer C–C bond length distances of 3, MFCTP were computed with B3LYP/6-311++G(d,p) compared to the other basis computation. The C–N bond distances have insignificant differentiations. Also, the O–H bond length vibrations were the shortest. The C–O bond length at B3LYP/6-311++G(d,p) was 1.382 Å and 1.373 Å at HF/6-311++G(d,p). The results of the B3LYP and HF techniques can be compared to see if the bond lengths are corrected or not.

**Table 2:** Equilibrium bond lengths (Å), bond angles (°) and dihedral angles (°) for the studied compound 3 (MFCTP) at the B3LYP/6-311++G(d,p) and HF 6-311++G(d,p) levels

Parameter	B3LYP/6-311++G(d,p)	HF 6-311++G(d,p)	Exp. X-ray^35,36^
**Bond length (Å)**			
C_30_–N_32_	1.385	1.367	1.388
C_26_=N_27_	1.319	1.307	1.290
C_30_–S_28_	1.823	1.811	—
C_7_=O_14_	1.225	1.216	1.207
C_11_–O_17_	1.382	1.373	1.384
N_32_–H_31_	1.011	1.002	—
C_7_–C_8_	1.478	1.466	1.521
C–H	1.083	1.078	0.933

**Bond angle (°)**			
<C_1_O_17_C_11_	106.27	105.75	117.61
<C_30_N_32_C_26_	117.29	116.92	119.71
<C_26_=N_27_C_9_	115.66	114.86	117.61
<C_9_O_16_C_3_	120.86	119.78	120.50
<O_29_C_30_N_32_	126.70	125.86	115.52
<N_32_C_26_N_27_	122.10	121.60	125.21
<N_27_C_9_O_16_	113.63	112.93	115.87
<O_29_C_30_S_28_	125.26	124.62	—
<C_30_S_28_C_13_	90.89	90.05	—
<O_14_C_7_C_4_	124.68	123.86	120.50
<S_28_C_13_C_12_	130.93	130.31	—

**Dihedral angle (°)**			
<C_4_C_5_C_6_C_10_	−178.87	−178.87	
<C_30_S_28_C_13_C_12_	179.75	179.75	
<O_29_C_30_S_28_C_13_	−179.99	−179.99	
<O_29_C_30_N_32_C_26_	−179.99	−179.99	
<N_32_C_26_N_27_C_9_	−179.99	−179.99	
<N_27_C_9_O_16_C_3_	−178.46	−178.46	
<C_18_O_15_C_5_C_6_	109.29	109.29	
<H_19_C_18_O_15_C_5_	66.183	66.183	

The angles of C_9_O_16_C_3_ (120.86°), O_29_C_30_N_32_ (126.70°), N_32_C_26_N_27_ (122.10°), O_29_C_30_S_28_ (125.26°), and O_14_C_7_C_4_ (124.68°) increased by DFT calculation, whereas the angle of N_27_C_9_O_16_ (113.63°) decreased from 120°. The steric and electronic effects resulting from the bond angle calculation illustrate that the ring of the molecule is distorted.

### Mulliken and actual natural charge scattering

**Fig. 7:**
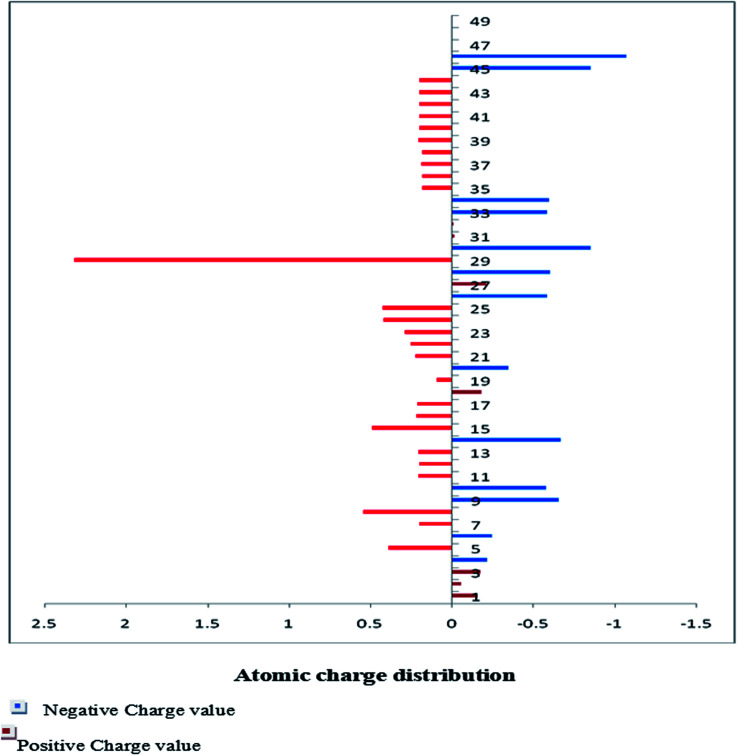
Mulliken atomic charge distribution (*e*) for the studied compound (3, MFCTP) at the B3LYP/6-311++G(d,p) level.

**Fig. 8:**
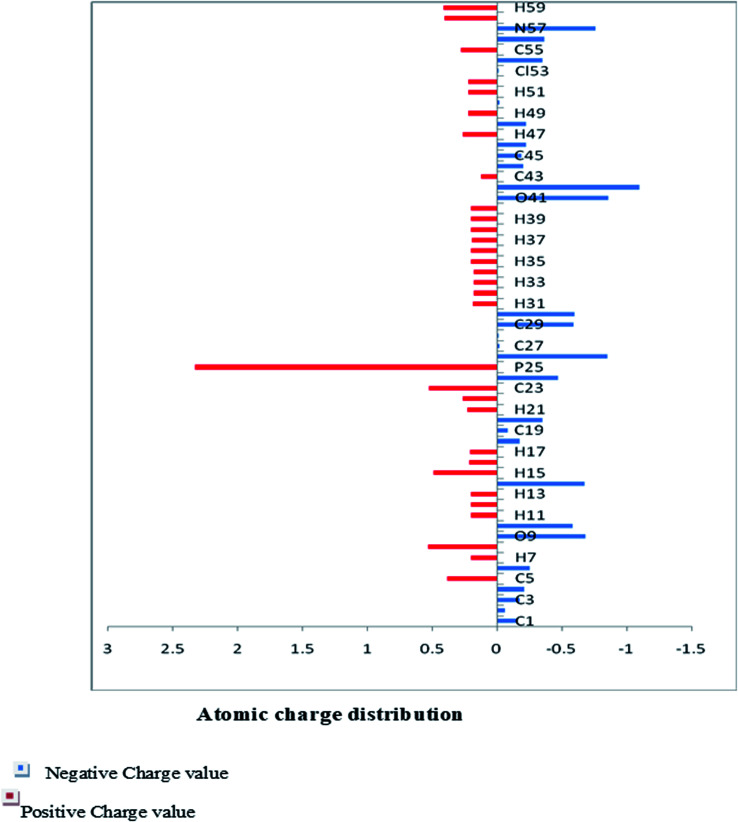
Natural atomic charge distribution (*e*) for the studied compound (3, MFCTP) at the B3LYP/6-311++G(d,p) level.

The Mulliken charge is shared among the close chemical bonds in the molecule (MFCTP), is openly linked to the vibrational parameters, and quantifies how the electronic structure loses charge under atomic dislocation. It modifies various properties of molecular systems such as dipole moment, polarizability, and electronic structure. Table 3 gives the computed Mulliken and natural charge values in the gaseous phase of the molecule (MFCTP) at the HF and B3LYP levels with various basis sets. Fig. 7 and 8 show the charge graphically and structural distribution of the Mulliken and natural charge of compound 3 (MFCTP), respectively.

**Table 3:** Mullikan and natural charges for the studied compound 3, MFCTP at the B3LYP/6-311++G(d,p) and HF 6-311++G(d,p) levels

Atom	Mulliken charges		Natural charges	
	B3LYP/6-311++G(d,p)	HF 6-311++G(d,p)	B3LYP/6-311++G(d,p)	HF 6-311++G(d,p)
N27	−0.229	−0.218	−0.487	−0.466
N32	−0.189	−0.125	−0.603	−0.590
O14	−0.273	−0.234	−0.588	−0.527
O15	−0.129	−0.102	−0.565	−0.536
O16	0.033	0.026	−0.482	−0.448
O17	−0.054	−0.045	−0.470	−0.427
O29	−0.270	−0.207	−0.563	−0.536
S28	−0.514	−0.456	0.306	0.295
H31	0.358	0.285	0.427	0.398
C6	0.584	0.485	−0.169	−0.139
C8	1.351	1.153	−0.241	−0.205
C26	0.054	0.045	0.406	0.386
C7	−0.258	−0.185	0.319	0.299
C4	0.799	0.689	0.574	0.545
C3	0.160	0.106	−0.143	−0.113
C12	−0.193	−0.139	−0.111	−0.101
C2	−0.479	−0.397	−0.284	−0.246
H22	0.248	0.184	0.254	0.225

All the N-atoms have the highest negative natural charges and the lowest Mulliken charges in all the basis sets, and the carbon atom fixed in the N-atoms is negative. In both the Mulliken and natural charges, all the hydrogen atoms are positive, except H31, which shows more deviations than the other H-atoms. H31 exhibit the maximum values for both charges of 0.358 and 0.427 at the B3LYP/6-311++G(d,p) level, respectively. This indicates that the substituted H-atom is more disturbed than the other hydrogen atoms.

### Thermodynamic studies

**Fig. 9:**
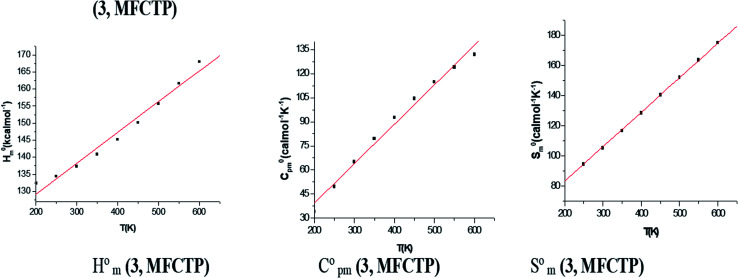
Correlation graphs of the thermodynamic properties and temperature for the studied compound (3, MFCTP) at the B3LYP/6-311++G(d,p) level.

Table 4 shows the ZPVE, thermal energy, specific heat capacity, rotational constants, and *S*_Vib_(*T*) entropy, where these thermodynamic parameters were calculated at room temperature of 298 K and 1.00 atm pressure using the B3LYP and HF methods with the 6-311++G(d,p) basis set for compound 3 (MFCTP). The rotational constant values decreased with the B3LYP method compared with the HF method. However, the mean difference between these two methods is only *ca.* 3 a.u. in global minimum energy. All the above-mentioned results were obtained without any proportional constrains. For B3LYP/6-311++G(d,p), it was found that the entropy values are higher than the other method. The total energies were found to decrease with an increase in the basis set dimensions, as shown by the ZPVE value, which is higher at 135.881 kcal mol^−1^ in B3LYP/6-311++G(d,p), but lower at 132.662 kcal mol^−1^ in HF/6-311G(d,p). Therefore, this indicates that the variation in the zero-point vibrational energies (ZPVEs) is significant, in addition to the lowest thermal energy of 145.341 mol^−1^ with HF/6-311G++(d,p) as the same global minimum energy. The vector of the dipole moment in three dimensions depends on the molecular charge distribution of a molecule and the center of positive and negative charges. The dipole moment value depends on the choice of origin and molecular orientation in charged systems and is strictly determined for neutral molecules. The heat capacity (*C*), entropy (*S*) and enthalpy changes (*H*) are standard statistical thermodynamic functions, which depend on the basis of the vibrational analysis at the B3LYP/6-311++G(d,p) level for the compound 3, MFCTP and obtained from the theoretical harmonic frequencies, as listed in Table 5. According to Table 5, the molecular vibrational intensities increase with temperature ranging from 200 to 600 K, which was observed from the thermodynamic functions.^40^ The quadratic formulas fitted the correlation equations for the heat capacities, entropy changes, and enthalpy with temperature and the *R*^2^ factors for the corresponding fitting are 0.99988, 0.99999 and 0.99988, respectively. Fig. 9 illustrates the correlation graphics, and the corresponding fitting equations are as follows:

1 *C*_p,m,__3__,__MFCTP_^0^ = 7.94349 + 0.21608*T* − 2.85319 × 10^−4^*T*^2^; (*R*^2^ = 0.99998),

2 *S*_m,__3__,__MFCTP_^0^ = 65.75467 + 0.18665*T* + 1.98708 × 10^−4^*T*^2^; (*R*^2^ = 0.99999),

3 *H*_m,__3__,__MFCTP_^0^ = 86.72153 − 0.05695*T* + 2.31806 × 10^−4^*T*^2^; (*R*^2^ = 0.99988),

**Table 4:** Calculated thermodynamic parameters for the studied compound 3 (MFCTP) at the B3LYP/6-311++G(d,p) and HF 6-311++G(d,p) levels

Parameter	B3LYP/6-311++G(d,p)	HF 6-311++G(d,p)
SCF energy (*E*_T_), (a.u.)	−1498.561	−1495.231
Zero point vibrational energy (kcal mol^−1^)	135.881	132.662
Rotational constant (GHz)		
*A*	0.65690	0.62472
*B*	0.10696	0.10478
*C*	0.09266	0.07355
Entropy total (*S*) (cal mol^−1^ K^−1^)	139.845	136.656
Translational	43.366	41.634
Rotational	35.156	32.145
Vibrational	61.323	60.233
Thermal energy total (*E*) (kcal mol^−1^)	147.403	145.341
Translational	0.889	0.889
Rotational	0.889	0.889
Vibrational	145.626	143.544
Specific heat (*C*_V_) (cal mol^−1^ K^−1^)	71.885	70.668
Translational	2.981	2.981
Rotational	2.981	2.981
Vibrational	65.923	62.894

**Table 5:** Thermodynamic properties at different temperatures of compound 3 (MFCTP) at the B3LYP/6-311++G(d,p) level

*T* (K)	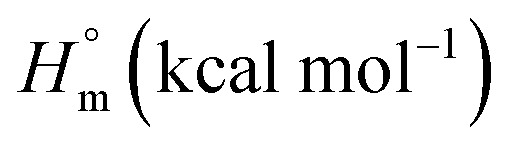	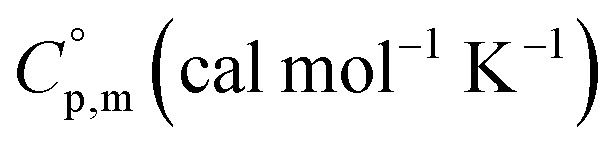	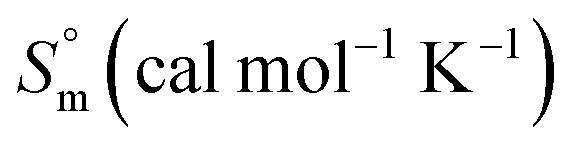
200	118.41	52.08	123.01
250	121.39	62.47	134.69
300	125.02	71.39	147.32
350	127.19	80.13	158.59
400	133.10	87.78	170.08
450	137.84	94.46	180.29
500	141.72	100.11	191.12
550	146.44	107.01	200.60
600	151.22	111.57	211.94

All the thermodynamic data supply helpful information for the further study of compound 3 (MFCTP). The second law of thermodynamics in the thermochemical field is used to compute the other thermodynamic energies according to relationships of thermodynamic functions and evaluation directions of chemical reactions, and it should be noted that all thermodynamic calculations were done in the gas phase and cannot be used in solution.

### Frontier molecular orbitals analysis (FMOs)

**Fig. 10:**
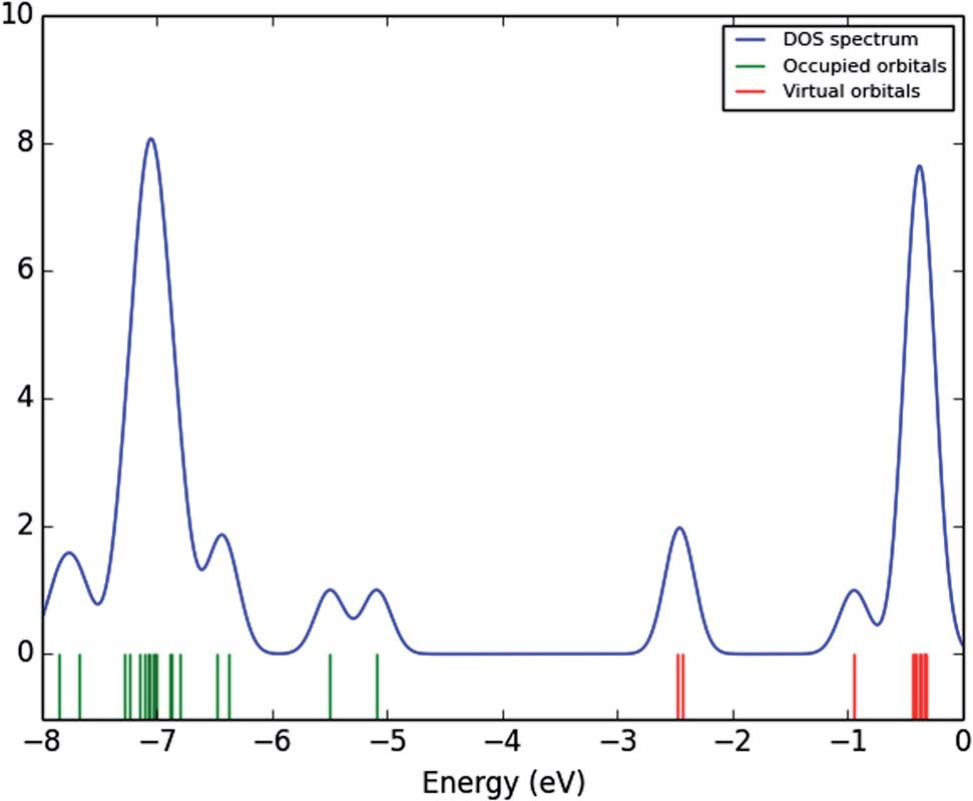
Density of states (DOS_s_) spectrum of (3, MFCTP).

The frontier molecular orbitals (FMOs) play an important role in the optical and electric properties, which are the (HOMO) and (LUMO), as well as in quantum chemistry and UV-vis spectra.^41^ The contributions of the molecular orbitals (HOMO and LUMO) were calculated using the Gauss-Sum 2.2 Program^42^ and Fig. 10 gives (DOS) the density of states. The HOMO has the ability to give an electron, whereas the LUMO obtains an electron as an electron acceptor. The kinetic stability, chemical reactivity, optical polarizability, and chemical hardness–softness of a molecule are determined by the energy gap between the HOMO and LUMO. These are expended as integral tools in the characterization of the chemical thermodynamic properties.

The FMO energy levels of the present compound MFCTP predict that an identical electronic displacement transition happens between the LUMO & HOMO, HOMO & LUMO + 1, and HOMO & LUMO + 2 orbitals, separately, and thus we performed calculations in DMSO, chloroform and the gas phase to estimate the energetic action of the title compound. Table 6 shows that for the S1 form of compound 3 (MFCTP), the energies of four important molecular orbitals were calculated using B3LYP/6-311++G as follows: the highest MOs (HOMO and HOMO − 1) and the lowest MOs (LUMO and LUMO + 1) (d,p). The calculated HOMO energy value is −6.3604, −6.3134 and −6.2843 eV in DMSO, chloroform and the gas phase, respectively. The LUMO energy is HOMO −2.5337, −2.5089 and −2.5013 eV, respectively. The energy gap value is 3.8267, 3.8045 and 3.7830 eV in DMSO, chloroform and gas phase, respectively.

**Table 6:** Calculated energy values, chemical hardness, electronegativity and dipole moment of compound 3 (MFCTP) in DMSO, chloroform and the gas phase at the B3LYP/6-311++G(d,p) level

Parameter	DMSO	Chloroform	Gas
Total energy (*E*_T_), (a.u.)	−1498.562	−1498.570	−1498.580
Energy of highest occupied molecular orbital (*E*_HOMO_) (eV)	−6.3604	−6.3134	−6.2843
Energy of lowest unoccupied molecular orbital (*E*_LUMO_) (eV)	−2.5337	−2.5089	−2.5013
Energy gap (*E*_g_) (eV)	3.8267	3.8045	3.7830
*E*_HOMO_ − 1 (eV)	−6.9708	−6.9336	6.9015
*E*_LUMO_ + 1 (eV)	−1.5406	−1.4887	1.4473
Energy gap (*E*_g_) (eV)	5.4302	5.4449	5.4541
*E*_HOMO_ − 2 (eV)	−7.3190	−7.3116	−7.3146
*E*_LUMO_ + 2 (eV)	−1.1078	−1.0730	−1.0505
Energy gap (*E*_g_) (eV)	6.2111	6.2386	6.2641
Dipole moment (*μ*)	4.4540	4.8964	5.3982
*I* (eV)	6.3604	6.3134	6.2843
*A* (eV)	2.5337	2.5089	2.5013
*X* (eV)	4.4470	4.4111	4.3928
*V* (eV^−1^)	−4.4470	−4.4111	−4.3928
*η* (eV)	1.9133	1.9022	1.8915
*S* (eV^−1^)	0.2613	0.2628	0.2643
*ω* (eV)	5.1678	5.1144	5.1009

**Table 7:** Experimental and computed excitation energies (in eV), electronic transition configurations, and oscillator strengths (*f*) for the optical transitions of the absorption bands in the UV-vis. regions (involving HOMOs) of the compound 3 (MFCTP) at the CAM-B3LYP/6-311++G(d,p)

Medium	Transition	Excitation energies	Type of transition	*λ*_max/nm_ Th and Ex.	Oscillator strength (*f*)	Configuration composition corresponding transition orbital
Gas phase	4	3.31	n–π*	374	0.0781	0.69(87–>88)
	9	3.97	n–π*	312	0.1917	0.10(85–>88); 0.66(86–>88)
	13	4.48	π–π*	277	0.3948	0.32(84–>88); 0.23(85–>88); 0.47(87–>89)
						0.27(87–>90); 0.13(87–>91)
	19	4.69	π–π*	264	0.3827	−0.17(82–>88); −0.21(84–>88); 0.15(84–>89); −0.16(85–>88); 0.10(85–>89); 0.56(89–>91)
	25	4.89	π–π*	254	0.1540	0.24(82–>88); 0.25(83–>88); 0.17(85–>89)
						0.13(86–>89); 0.54(87–>91)
	34	5.18	π–π*	239	0.1167	0.43(84–>89); 0.24(85–>89); 0.39(86–>90)
						−0.17(87–>90); −0.12(87–>91)
DMSO	3	3.26	n–π*	380	0.1006	0.70(87–>88)
				493		
	9	3.91	n–π*	317	0.3050	0.69(86–>88)
				350		
	13	4.45	π–π*	279	0.6216	−0.13(84–>88); 0.39(85–>88); 0.51(87–>89)
				300		
	17	4.66	π–π*	266	0.4791	−0.20(85–>88); 0.14(85–>89); 0.61(87–>90)
				270		
	24	4.88	π–π*	254	0.0962	0.15(82–>88); 0.11(83–>88); 0.29(86–>89)
				240		−0.10(87–>90); 0.59(87–>91)
	40	5.53	π–π*	225	0.0879	−0.12(83–>89); 0.27(84–>90); −0.36(86–>91)
				200		0.10(86–>93); −0.47(87–>93)
Chloroform	4	3.27	n–π*	379	0.1134	0.69(87–>88)
				477		
	9	3.92	n–π*	316	0.3198	0.10(85–>88); 0.69(86–>88)
				349		
	13	4.42	π–π*	280	0.7212	−0.24(84–>88); 0.36(85–>88); 0.51(87–>89)
				302		
	18	4.65	π–π*	267	0.4068	−0.15(85–>88); 0.13(85–>89); 0.62(87–>90)
				275		
	24	4.87	π–π*	256	0.0893	0.52(82–>88); 0.26(83–>88); 0.11(85–>89)
				265		
	33	5.17	π–π*	240	0.1011	−0.38(84–>89); 0.36(85–>89); 0.39(86–>90)
				250		

**Fig. 11:**
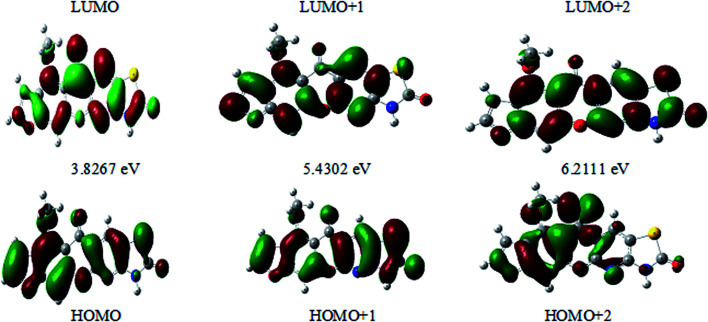
Molecular orbitals and energies for the HOMO and LUMO of (3, MFCTP) in the gas phase.

The HOMO–LUMO energy gap indicates the final charge transfer interaction within the molecule, which influences the biological activity of the current compound. Additionally, the bandgap narrows as one transitions from the gas phase towards a suitable solvent. At the B3LYP/6-311++G(d,p) level, 3D plots of the HOMO and LUMO orbitals were computed for compound 3 (MFCTP), as shown in Fig. 11 (in the gas phase). The HOMO–LUMO transition displays an electron density assignment, and thus the HOMO is found throughout the molecule, particularly on the rings, whereas the LUMO is only found on the ring and the O-atom. These can be observed in the figure, where the positive phase is red and the negative phase is green.

The values of electronegativity, chemical hardness, softness, and electrophilicity index for compound MFCTP are 4.4470, 1.9133, 0.2613 and 5.1678 eV in DMSO, respectively. These values are greater compared to that in chloroform and the gas phase. The dipole moment in a molecule is another important electronic property. For example, the bigger the dipole moment, the stronger the intermolecular interactions. The calculated dipole moment values for the molecules are also presented in Table 6. Based on the predicted dipole moment values, it was found that, going to the solvent phase (4.4540 D in DMSO and 4.8964 D in chloroform) from the gas phase (5.3982 D), the dipole moment value increases (Table 6).

### Electrochemical potential, total negative electrons, and transport of electrons in the molecule

**Fig. 12:**
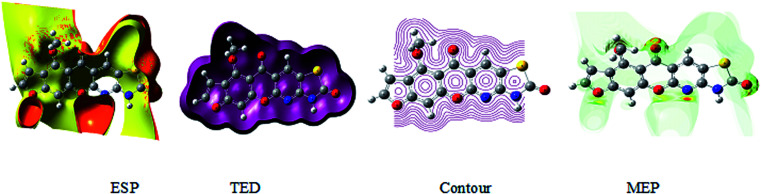
Electrostatic potential (ESP), total electron density (TED), contour and the molecular electrostatic potential (MEP) map in the gas phase of (3, MFCTP).

The 3D plot of the electrostatic potential (ESP) and total electron density (TED) plots show a uniform distribution and molecular electrostatic potential (MEP) map of 3, MFCTP, as illustrated in Fig. 12. However, the ESP negative plot is more focused on the N- and O-atoms in the molecule and is returned as a yellowish blob owing to a proton attracted either by the concerted electron density of the particle (red color on the ESP surface) or the repulsion of the proton by atomic nuclei followed by the positive electrostatic potential in regions where the nuclear charge is incompletely shielded (and shades of blue of color) at low electron density. This result is expected because the ESP correlates with the electronegativity and partial charges. The interest in the MEP lies in the fact that it is useful in the research of molecular structure due its physiochemical property relationship^43–45^ and it also displays molecular size, shape and positive or negative (Fig. 12).

The different colors represent the different values of the electrostatic potential at the surface. The potential increases in the order of red < orange < yellow < green < blue. Blue indicates the strongest attraction and red indicates the strongest repulsion, where these maps have a color code that varies between −0.06079 a.u. (darkest red) through 0.06079 a.u. (darkest blue) inside the compound. The negative potential sites are on the nitrogen atoms and over the ring carbon and sulfur atoms, whereas the potential positive sites are around the H-atom in the amino group (–NH), as shown in the MEP map of the title molecule. The front and side views of the methoxy group in 3, MFCTP have a green color, indicating a neutral potential.

### NLO properties

The mean polarizability, 〈*α*〉, anisotropy of the polarizability, Δ*α*, total static dipole moment, *μ*, and the mean first polarizability, 〈*β*〉 using the *x*, *y*, *z* components were calculated using the B3LYP/6-311++G(d,p) basis set from the Gaussian 09 W output, as cited in the literature.^46^ Among the second order NLO parameters, we focused on the hyper-Rayleigh scattering (*β*_HRS_) and depolarization ratio (DR),^46^ which are calculated as follows:4
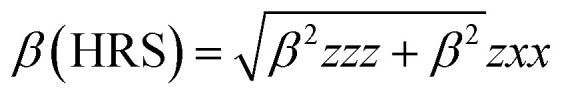
5
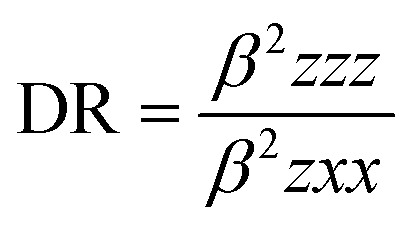


The deviation in the amounts of *β*_HRS_ and DR can be recognized by the structural index.^47^

The ability of any compound to convert light [with intense electric field (LASER)] of longer wavelength into light of shorter wavelength is known as non-linear optical properties. The second harmonic generation (SHG) is a non-linear optical phenomenon in which intense light with a longer wavelength is converted to half of the incident value after absorption by a non-linear optical material, as shown in the figure below.

### Second harmonic generation

Table 8 lists the above-mentioned computed values, as well as the electronic dipole moment {*μ*_*i*_ (*i* = *x*, *y*, *z*) and total dipole moment *μ*_tot_} for the title compound. The continuity formula was used to collect sufficient polarity.*μ* = (*μ*_*x*_^2^ + *μ*_*y*_^2^ + *μ*_*z*_^2^)^1/2^

**Table 8:** Total static dipole moment (*μ*), mean polarizability (〈*α*〉), anisotropy of the polarizability (Δ*α*), and mean first-order hyperpolarizability (〈*β*〉) for compound 3 (MFCTP) at the B3LYP/6-311++G(d,p) level

Property	(3, MFCTP)
*μ*_*x*_, D	−4.2260
*μ*_*y*_, D	−0.7061
*μ*_*z*_, D	1.2166
*μ*, debye^a^	4.4539
*α*_*xx*_, a.u.	−147.8995
*α*_*xy*_, a.u.	0.4812
*α*_*yy*_, a.u.	−142.1829
*α*_*zz*_, a.u.	−143.8694
*α*_*yz*_, a.u.	4.1951
*α*_*xz*_, a.u.	−3.0669
〈*α*〉 × 10^−24^ esu	35.913
Δ*α* × 10^−24^ esu	45.750
*βxxx*, a.u.	−396.4089
*βxxy*, a.u.	16.0264
*βxyy*, a.u.	8.9499
*βyyy*, a.u.	−4.9009
*βxxz*, a.u.	3.5689
*βxyz*, a.u.	−14.8281
*βyyz*, a.u.	12.7146
*βxzz*, a.u.	8.9966
*βyzz*, a.u.	0.8604
*βzzz*, a.u.	1.5237
〈*β*〉 × 10^−30^ esu^a^	3.6781
DR	0.46
*β*_HRS_	56.12

a Urea equal (1.3197 D, 0.1947 esu) results are taken from ref. 50.

It is well known that higher values of the dipole moment, molecular polarizability, and hyperpolarizability are important for more active NLO properties. The polarizabilities and hyperpolarizability are reported in atomic units (a.u.), and the calculated values were converted into electrostatic units (esu) (for *α*; 1 a.u. = 0.1482 × 10^−24^ esu, for *β*; 1 a.u. = 8.6393 × 10^−33^ esu). The computed dipole moment (*μ*) value was 4.4539 debye. For component *μ*_*x*_, the largest dipole moment was observed. This value is equal to −4.2260 D in this direction. Compound 3 (MFCTP) has a computed polarizability of 35.913 × 10^−24^ esu. One of the most essential variables in an NLO system is the size of the molecule hyperpolarizability, *β*. The computed initial hyperpolarizability value (*β*) of compound 3 (MFCTP) by B3LYP/6-311++G(d,p) is 3.6781 × 10^−33^ esu.^48^ Also, for the examined molecule, the lowest value of *β* and DR, and the greatest value of *β*_HRS_ confirm its short bond length, indicating better selectivity. Consequently, the process of converting solar energy changed in Table 9. When evaluating the computational error percentage, the values of the derived parameters for compound 3 (MFCTP) were close to the values found for similar structures, confirming the accuracy of the obtained results.^49–52^ Urea is one of the classic compounds utilized in the research of the NLO characteristics of molecular systems. Thus, it was employed as a comparative threshold value. The dipole moment of compound 3 (MFCTP) is larger than that of urea, although the initial hyperpolarizability of the title molecule is bigger than that of urea (*μ* and *β* of urea are 1.3197 debye and 0.1947 × 10^−33^ esu, respectively, obtained using the B3LYP/6-311++G(d,p) technique).^53^

**Table 9:** Comparative analysis of compound 3 (MFCTP) with similar types of compounds

Parameter	3, MFCTP	Ref. 46	Ref. 47	Ref. 48	Ref. 49
Energy of highest occupied molecular orbital (*E*_HOMO_)	6.3604	−6.556	−4.89	−6.4355	−6.087
Energy of lowest unoccupied molecular orbital (*E*_LUMO_)	2.5337	−2.536	−1.496	−2.3147	−1.824
Energy gap (*E*_g_)	3.8267	4.020	3.393	4.1208	4.263
Dipole moment (*μ*)	4.4540	7.8870	6.5231	7.2563	7.8521
*I* (eV)	6.3604	6.55602	4.89	6.4355	6.087
*A* (eV)	2.5337	2.53586	1.496	2.3147	1.824
*X* (eV)	4.4470	4.54594	3.193	4.3751	3.955
*V* (eV^−1^)	−4.4470	−4.54594	−3.193	−4.3751	−3.955
*η* (eV)	1.9133	2.01008	1.6965	2.0604	2.1315
*S* (eV^−1^)	0.2613	0.24875	0.29472	0.24267	0.23458
*ω* (eV)	5.1678	5.14048	3.004789	4.645093	3.669253

### Vibrational calculation characteristics

**Fig. 13:**
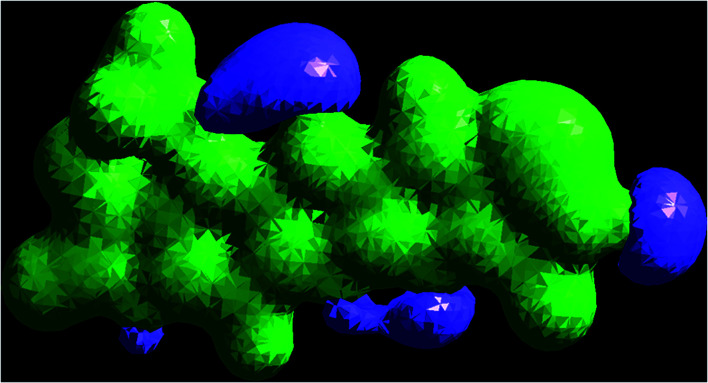
3D potential energy distribution map of (3, MFCTP).

The comparison with the theoretical PED-scaled wavenumbers by the B3LYP methods was based on the normal mode analyses of the experimental vibrational wavenumber assignments. Considering that density functional theory includes the electron correlation HF, but has a lower frequency value, each one of the PEDs from this data set is discussed in detail, and the distribution is shown Fig. 13.

Because the computations were performed in the gaseous state, repulsion between the theoretical and observable vibrations occurred. The estimated vibrational frequencies at the DFT/B3LYP/6-311++G (d,p) level and the experimental values whose associated assignments from the FT-IR spectra of compound 3 (MFCTP) produce an unscaled calculated frequency are shown in Fig. 1 and Table 10.

**Table 10:** Experimental and computational calculated vibrational wavenumbers (harmonic frequency (cm^−1^)), (scaled and unscaled values), IR intensities and assignments for compound 3 (MFCTP) at the B3LYP/6-311++G(d,p) level

No.	Exp.	Wavenumber		IR intensity		Assignments	References
		Unscaled	Scaled	Rel.	Abs.		
1	3225	3310	3210	86.67	41.75	*ν*N–H	51
2	3122	3220	3120	55.58	21.57	*ν*C–H_furan_	51
3	3055	3170	3047	21.84	12.84	*ν*C–H_aromatic_	51
4	2948, 2841	2960, 2900	2910, 2820	26.22	16.72	*ν*C–H_aliphatic_	51
5	1638	1750	1650	119.62	87.35	*ν*C=O_γ-pyrone_	52
6	1679	1790	1685	21.47	45.26	*ν*C=O_cyclic amide_	52
7	1568	1650	1585	107.25	52.16	*β*C=C_(in ring)_	52

a *ν* twisting (stretching); *ν*_2_ (symmetric stretching); *ν*_3_ (asymmetric stretching); *β* (in plane bending).

To make up for the methodological flaws caused by basis set incompleteness, default electron correlation, and vibrational harmonics, a spectrum uniform scaling factor was utilized to perfect the computed values in agreement with the observed values. Consequently, the vibrational frequencies estimated at the B3LYP/6-311++G(d,p) level were scaled by 0.9613. With a slight exception, the deviation from the experiments was less than 10 cm^−1^ after scaling with the calibration factor. How this task can be carried out on a large scale is described as follows.

At 3225 cm^−1^, the observed N–H vibration twisting was feasible, similar to the estimated vibration at 3310 cm^−1^ (unscaled value) and 3210 cm^−1^ (scaled value). Furthermore, the experimental C–H_furan_ stretching vibration was found to be symmetric at 3122 cm^−1^, which is consistent with the estimated vibrations at 3220 cm^−1^ (unscaled value) and 3120 cm^−1^ (scaled value). The aromatic expansion vibrations (C–H)^54^ emerged in the range of 3000–3100 cm^−1^, with the estimated vibration of the C–H aromatic at 3170 cm^−1^ (unscaled value) and 3047 cm^−1^ (scaled value) and empirically detected at 3055 cm^−1^. Furthermore, the C–H symmetric aliphatic stretching vibration in CH_3_ was measured experimentally at 2948 and 2841 cm^−1^ and estimated at 2960 and 2900 cm^−1^ (unscaled value) and 2910 and 2820 cm^−1^ (scaled value), for compound (3, MFCTP), respectively. In general, the C=O vibrations were recorded in the range of 1790–1810 cm^−1^,^55^ with experimental values of 1638 cm^−1^ and 1679 cm^−1^, and theoretical values of 1750 cm^−1^ and 1790 cm^−1^ (unscaled value) and 1650 cm^−1^ and 1685 cm^−1^ (scaled value) for compound (3, MFCTP), respectively. C=C vibrations are commonly detected in the range of 1480–1630 cm^−1^,^56^ and experimentally recorded at 1568 cm^−1^, with computed vibrations at 1650 cm^−1^ (unscaled value) and 1585 cm^−1^ (scaled value).

### UV-spectra in electronic form

**Fig. 14:**
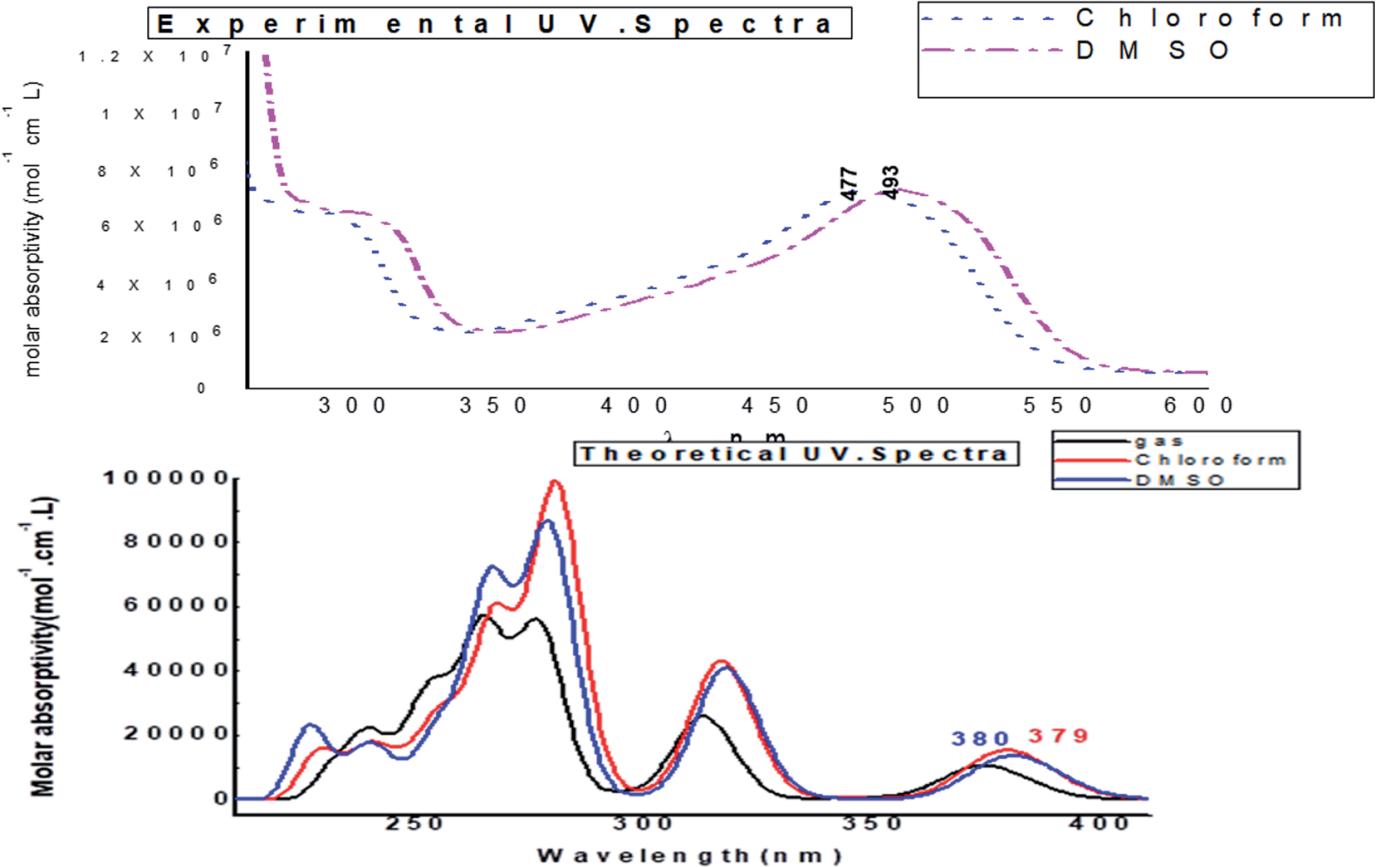
Experimental UV-vis. spectra and calculated electronic “absorbance” spectra of the studied compound (3, MFCTP) in different solvents.

**Fig. 15:**
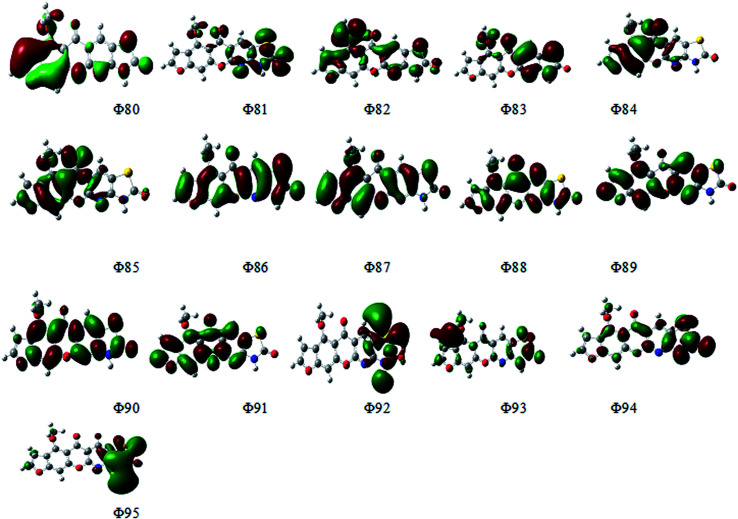
Electron density contours of compound (3, MFCTP).

Fig. 14 depicts the effect of the solvent on the electronic spectrum of compound (3, MFCTP) for both the theoretical and experimental data. The charge density maps of the occupied and unoccupied MOs are shown in Fig. 15. Six bands of non-polar solvent (chloroform) were found in the spectrum at 477, 349, 302, 275, 265, and 250 nm. When gradually increasing the polarity of the solvent to DMSO, the range of spectrum bands for the excited and ground states has the same values; additionally, the strength of the bands increases with polar solvents, and thus all the bands shift to (π–π*) and (n–π*). The electrification causing a conflict between theoretical and experimental results is created from the electron excitation of the ten MO molecular orbitals φ_82_^−1^φ_88_–φ_89_^−1^φ_98_ for MFCTP. The first (n–π*)^1^ band in a non-polar solvent (chloroform) was seen at 477 nm and theoretically at 379 nm through a configuration of φ_87–88_^−1^, as shown in Table 7 and Fig. 15. Conversely, in the polar solvent (DMSO), the band at 493 nm corresponds to the computed band at 380 nm. The electron density characteristics are derived from the nature of the molecular orbital, according to the electronic transformation. The delocalization of the electron density and the charge transfer characteristic can be seen in Fig. 15. In the visible area, the absorption bands are the typical n–π* and π–π* transitions.

### Analysis of natural bond orbitals (NBO)

The synthesized compound (3, MFCTP) was investigated using the natural bond orbital method (NBO),^57^ and the molecular electrostatic potential (MEP) was calculated. The second order Fock matrix for each donor (*i*) and acceptor (*j*) was calculated using the donor–acceptor interactions and the stabilization energy *E*^(2)^ associated with the delocalization *i* → *j* was determined as follows:

6 *E*^(2)^ = Δ*E*_*ij*_ = *q*_*i*_(*F*(*ij*)^2^/*ε*_*j*_ − *ε*_*i*_),

where *q*_*i*_ is the donor orbital occupancy, *ε*_*i*_, and *ε*_*j*_ are the diagonal elements, and *F*(*ij*) is the off-diagonal NBO Fock matrix element. The higher the *E*^(2)^ value, the stronger the contact between the donor and acceptor orbitals, and therefore the delocalization trend of electrons. Table 11 shows the NBO of the optimal structures that were chosen. Higher values of the stabilization energies for the πC3–C4 → RY*N27, πC11–H25 → RY*C6, πC9–N27 → RY*H23, πC3–C4 → RY*C30, πC3–C4 → π*C1–C2, πC1–O17 → RY*N27, and πC1–O17 → π*C18–H20 interactions were achieved in geometry of compound (3, MFCTP). The strong lone pair-anti-bonding orbital interactions between the two ends of the generated H-bond account for the substantial stabilization effects. The structure of compound 3 (MFCTP) has a stronger H-bond with the methoxy group based on these findings. Consequently, the structure of MFCTP is more stable.

**Table 11:** Significant *E*^(2)^ values (kcal mol^−1^) for the optimized structure 3, MFCTP of selected interactions in the NBO analysis at the B3LYP/6-311++G(d,p) level

Charge transfer	*E* ^(2)^ a (kcal mol^−1^)	NBO	Population
πC3–C4 → RY*C30	27 657.25	πC3–C4	1.92482
πC3–C4 → RY*O16	36 937.61	πC1–O17	1.99011
πC3–C4 → π*C1–C2	8813.97	πC2–C3	1.68583
πC1–O17 → σ*C9–S28	29.81	πC9–N27	1.97797
πC1–O17 → RY*C4	8229.84	πC11–H25	1.98003
πC1–O17 → RY*N27	9709.72	σC16–S28	1.97540
πC1–O17 → RY*H22	10 068.96	CR C13	1.99878
πC1–O17 → RY*S28	22 486.29	CR O15	1.99969
σC1–O17 → RY*O29	2441.01	CR N27	1.99922
πC1–O17 → π*C2–C2	15 214.44	CR S28	1.99931
πC1–O17 → σ*C30–N32	5085.36	LP N27^b^	1.89133
πC1–O17 → σ*C18–H20	481.73	LP S28^b^	1.98423
πC2–C3 → RY*O29	86.97	LP O29^b^	1.97629
πC2–C3 → RY*H31	5969.53	LP N32^b^	1.64200
πC3–C4 → RY*N27	7309.64	π*C1–C6	0.47477
πC3–C4 → RY*S28	927.47		
πC3–C4 → RY*O29	4198.24		
πC3–C4 → πC2–H23	3667.05		
πC3–C4 → π*C30–O29	287.54		
πC3–C4 → π*C30–N32	1938.51		
πC3–C4 → σ*C30–S28	1153.84		
πC9–N27 → RY*C11	665.09		
πC9–N27 → RY*O16	486.39		
πC9–N27 → RY*H23	521.83		
πC9–N27 → RY*N27	41.58		
πC9–N27 → RY*S28	60.56		
πC9–N27 →π*C9–N27	10.89		
πC9–N27 → πC18–H20	226.02		
πC9–N27 → π*C30–O29	18.17		
πC9–N27 → πC10–C11	40.78		
πC11–H25 → RY*C6	104.57		
σC16–S28 → RY*C2	3376.61		
CR C13 → RY*C30	554.67		
CR O15 → RY*C13	7494.25		
CR N27 → RY*C9	2355.06		
CR S28 → RY*C9	254.32		
CR O29 → RY*N32	8232.90		
LP N27 → RY*C6^b^	1629.32		
LP S28 → RY*C9^b^	1425.62		
LP O29 → RY*S28^b^	1155.36		
LP N32 → RY*H31^b^	1248.35		
π*C1–C6 → RY*C8	3600.21		

a *E* ^(2)^ means energy of hyper-conjugative interactions (stabilization energy).

b LP_(*n*)_ is a valence lone pair orbital (*n*) on an atom.

### Natural population analysis and natural charges

Table 12 shows the natural electronic configuration of the MFCTP active sites at the B3LYP/6-311++G(d,p) level, as well as the natural charge and population of total electrons on the subshells. The more positive the atom, the more likely it is to accept an electron. The O14, O15, O16, O17, N27, S28, O29, and N32 atoms had the most negative center atoms, implying an electron is shielded from the static electricity of the MFCTP molecule. Furthermore, in the natural population analysis, MFCTP has 174 electrons that are coordinated in sub-shells as a total Lewis and a total non-Lewis structure.

**Table 12:** Natural charge and natural population analysis for compound 3 (MFCTP) at the B3LYP/6-311++G(d,p) level^a^

Atom no.	Natural charge	Natural population				Natural electronic configuration
		Core	Valence	Rydberg	Total	
O14	−0.58761	1.999	6.5732	0.01468	8.588	[Core]2S (1.70)2p (4.88)3p (0.01)
O15	−0.56536	1.999	6.5405	0.02522	8.565	[Core]2S (1.59)2p (4.95)3p (0.01)
O16	−0.48162	1.999	6.4599	0.02202	8.482	[Core]2S (1.57)2p (4.89)3p (0.01)
O17	−0.47021	1.999	6.4543	0.01622	8.470	[Core]2S (1.60)2p (4.86)3p (0.01)
N27	−0.48670	1.999	5.4674	0.02007	7.487	[Core]2S (1.35)2p (4.11)3p (0.01)
S28	−0.30589	9.999	5.6581	0.03697	15.694	[Core]3S (1.69)3p (3.97)3d (0.02)
O29	−0.56310	1.999	6.5463	0.01708	8.563	[Core]2S (1.69)2p (4.86)3p (0.01)
N32	−0.60349	1.999	5.5891	0.01521	7.603	[Core]2S (1.26)2p (4.33)4p (0.01)

a Core 55.97789 (99.961% of 56). Valence Lewis 112.99443 (95.758% of 118). Total Lewis 168.97233 (97.111% of 174). Valence non-Lewis 4.55012 (2.615% of 174). Rydberg non-Lewis 0.47756 (0.274% of 174). Total non-Lewis 5.02767 (2.889% of 174).

### Antimicrobial properties

**Fig. 16:**
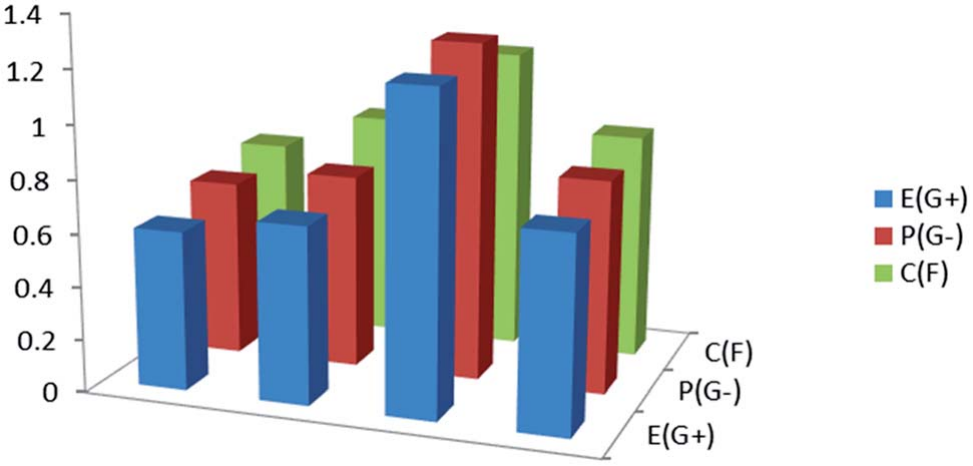
Antimicrobial activity for the studied compound (3, MFCTP) against Gram-positive bacteria (G^+^), Gram-negative bacteria (G^−^), and fungi (F).

The biological activities of the synthesized compound (3, MFCTP) were studied to determine it antibacterial and antifungal properties against different types of bacteria including Gram-positive: *S. aureus* and *B. subtilis* and Gram-negative: *S. typhimurium* and *E. coli*, and yeast: *C. albicans* and fungus *A*. *fumigatus*. The results of the growth inhibition (zone of inhibition) surrounding the disc of material and shown in Table 13 and Fig. 16. Some antibiotics were evaluated for their antibacterial activities and their results were found to be ineffective against all the tested bacteria and fungus. Compound (3, MFCTP) exhibited high antimicrobial activity against all the tested bacteria and fungi, in which the small size of compound (3, MFCTP) increases its absorption ability on the surface of the cell wall of microorganisms and the respiration process of the cell. Hence, compound (3, MFCTP) exhibits a growth-inhibitor effect.

**Table 13:** *In vitro* antimicrobial activities of the synthesized compounds at 500 and 1000 μg mL^−1^ and the MIC values for compound 3 (MFCTP)

Compd	Conc. (μg mL^−1^)	Zone of inhibition in mm^a^ and (MIC values in μg mL^−1^)					
		Bacteria Gram (+)ve		Bacteria Gram (−)ve		Yeast	Fungi
		*S. aureus*	*B. subtilis*	*S. typhimurium*	*E. coli*	*C. albicans*	*A. fumigatus*
MFCTP	500	—	—	—	—	12	—
	1000	—	—	—	—	16	—
S^b^	500	26	25	28	27	28	26
	1000	35	35	36	38	35	37

a Low active: 6–12 mm; moderately active: 13–19 mm; highly active: 20–30 mm; —: no inhibition or inhibition less than 5 mm.

b S: standard drugs.

### Structure–activity relationship (SAR)

The biological activity of the prepared compound (3, MFCTP) can be correlated with its calculated ground state energetic and global properties. According to Tables 6 and 13, the biological activity of the studied compound (3, MFCTP) obtained experimentally shows that it is highly active against G+, G− and fungi. Theoretically, the chemical reactivity can be explained in terms of the energy gap, *E*_g_, of the studied compound computed at the B3LYP/6-311++G(d,p) level, which follows the same activity trend obtained experimentally, indicating that *E*_g_ is one factor contributing to the reactivity of the studied compound (3, MFCTP). Also, *E*_HOMO_, which measures the donating power, and the dipole moment, which measure the charge separation, (*cf.*Table 6) can be employed to explain the activity. The theoretically computed global softness (*S*), global electrophilicity index (*ω*), electronegativity (*χ*), and chemical potential (*V*) of the studied compound (3, MFCTP) follow the same experimental biological activity. In contrast, the chemical hardness (*η*) follows the opposite experimental biological activity. In the case of natural charge from NBO and mean first-order hyperpolarizability (*β*), they violate the order of the experimental biological activity. In conclusion, the substituent in compound (3, MFCTP) increases its biological activity.

## Conclusion

The novel annulated 10-methoxy-10*H*-furo[3,2-*g*]chromeno[2,3-*b*][1,3]thiazolo[5,4-*e*]pyridine-2,10(3*H*)-dione (3, MFCTP) was efficiently synthesized from ring-opening, ring-closure reactions of 4-methoxy-5-oxo-5*H*-furo[3,2-g]chromene-6-carbonitrile (1) with 1,3-thiazolidine-2,4-dione (2) in boiling ethanol containing piperidine. The HF and DFT-B3LYP methods with the 6-311++G(d,p) basis set were used to perform a full vibrational study of MFCTP. In the DFT/B3LYP/6-311++G(d,p) technique, the observed and stimulated spectra agree for a good frequency fit. The structure and symmetry features of the title molecule were determined using a variety of quantum chemical computations. Theoretical calculations were used to examine the UV-vis spectra of MFCTP. For the electronic absorption spectra in the gas phase and solvent, TD-DFT calculations were performed to understand the electronic transitions of the current compound MFCTP (DMSO and chloroform). According to NLO study, the produced molecule offers good benefits in technology-related applications compared to the urea molecule. Negative potential sites are found around its oxygen and nitrogen atoms, while positive potential sites are found on its hydrogen atoms, according to the ESP and MEP maps. The chemical shift analysis from the NMR study confirmed the charges predicted by MEP. The most likely transitions in the compound were found using NBO analysis. In addition, its thermodynamic properties were computed. We also found relationships between the statistical thermodynamics and temperature. Because the intensities of the molecular vibrations increase with an increase in temperature, the heat capacity, entropy, and enthalpy all increase. Furthermore, compound (3, MFCTP) exhibited high antimicrobial activity against all the tested bacteria and fungi.

## Conflicts of interest

There are no conflicts to declare.

## Supplementary Material
